# Impact of Decreased Night Work on Workers’ Musculoskeletal Symptoms: A Quasi-Experimental Intervention Study

**DOI:** 10.3390/ijerph17239092

**Published:** 2020-12-05

**Authors:** Hye-Eun Lee, Min Choi, Hyoung-Ryoul Kim, Ichiro Kawachi

**Affiliations:** 1 Korea Institute of Labor Safety and Health, Seoul 07023, Korea; minchoi2015@gmail.com; 2 Department of Social and Behavioral Sciences, Harvard T.H. Chan School of Public Health, Boston, MA 02115, USA; ikawachi@hsph.harvard.edu; 3 Department of Occupational and Environmental Medicine, College of Medicine, The Catholic University of Korea, Seoul 06591, Korea; cyclor@catholic.ac.kr

**Keywords:** night shift work, musculoskeletal, difference-in-difference, organizational intervention, South Korea

## Abstract

A possible association between night shift work and musculoskeletal disorder has been suggested. This study aimed to evaluate the impact of decreased night work on musculoskeletal pain. Difference-in-difference estimation was used to compare changes in musculoskeletal pain between shift workers (*N* = 122) and non-shift workers (*N* = 170) in a manufacturing company before and after the introduction of a new shift system eliminating overnight work. Musculoskeletal pain was measured by a questionnaire asking if workers had symptoms in specific body parts, including the neck, shoulder, arm/elbow, wrist/hand, back, and leg/foot, over the past year. Generalized estimating equation models were used to estimate changes in pre- versus post-intervention musculoskeletal pain rates between the treated and control group. In the difference-in-difference (DID) models, prevalence of musculoskeletal pain for shoulder (−10.3%), arm (−12.9%), all sites combined (−9.2%), and upper extremity combined (−14.8%) showed significant decreases from pre- to post-intervention among the treated group (shift workers) compared to the control group (non-shift workers) after controlling for age and weekly working hours. Decreasing night work was related to improvement in musculoskeletal pain in shift workers.

## 1. Introduction

Night shift work has been reported to be associated with adverse health outcomes, including disturbed sleep, obesity, depression, heart disease, and cancer [1]. Exposure to “shift work” is common in modern society, and workers who work “non-standard working hours” are increasing worldwide. Around 19% of the workers in 28 European countries responded that their working hour arrangement involved night work [2]. In South Korea, approximately 10% of Korean workers were engaged in shift work, which is lower than in Western settings. However, the most common shift type (50.4%) was rotating two groups with two shifts, under which the workers usually work for 12 or 24 h per shift [3]. Considering that Korea has the some of the longest working hours in the Organisation for Economic Co-operation and Development (OECD) countries, shift workers in Korea are likely to be exposed to a higher intensity of night work, i.e., frequency and duration of night shifts.

Several studies have reported night work to be associated with increased risk of musculoskeletal diseases or pain [4,5,6,7]. The association between night work and musculoskeletal diseases is possibly related to sleep deprivation. Sleep problems have been shown to increase the risk of musculoskeletal complaints in several prospective studies [8,9]. Insufficient recovery due to reduced or poor sleep can be on the pathway between night work and musculoskeletal symptoms. Work-related musculoskeletal diseases account for a remarkable proportion of occupational health problems, consisting of around 70% of compensated occupational diseases in Korea [10]; moreover, the costs for work-related musculoskeletal diseases in the USA were USD 2.6 billion [11]. Therefore, numerous studies have been performed to identify risk factors for work-related musculoskeletal diseases in order to suggest potential interventions to prevent these problems. However, previous studies focusing on night work and musculoskeletal problems were mostly cross-sectional studies [4,5,7,12], while a few studies were longitudinal [6,13]. Intervention studies in this area remain scarce.

In 2013, a major automobile manufacturer in Korea, the Hyundai Motor Company, introduced a system of two consecutive shifts per day, putting an end to the overnight shift system for the 24 h operation that had prevailed for 46 years since the opening of the plant [14]. Other automobile manufacturers and related automobile parts manufacturers in Korea followed the lead of Hyundai, and a number of workers have benefited from reduced working hours and elimination of overnight work.

In the present study, the Doowon Precision Industry Co. (hereafter, Doowon), an automobile engine pump manufacturer, introduced a new shift system in 2010 after negotiations with the labor union. This change of working hour arrangements enabled us to evaluate the impact of decreased night work by observing the workers’ musculoskeletal pain before and after the policy change. Our difference-in-difference (DID) estimation attempts to mimic the approach of a randomized controlled trial [15]. Therefore, the purpose of this study is to investigate the impact of decreased night work on musculoskeletal pain using a quasi-experimental design.

We hypothesized that musculoskeletal pain in the treatment group (i.e., shift workers who experienced a decreased night work) would be improved compared to the control group (i.e., daytime workers who experienced no change in night work) following the change in the shift system. 

## 2. Materials and Methods

### 2.1. Study Design

We used a difference-in-difference (DID) estimation to compare the change in the prevalence of musculoskeletal pain among shift workers and non-shift workers before and after the introduction of the new shift system. On 30 September 2010, Doowon introduced 2 consecutive shifts (day and evening) per day instead of rotating 2 groups with 2 shifts (day and night). Previously, the first shift began at 8:30 a.m. and ended at 7:30 p.m., with the second shift running from 7:30 p.m. to 6:20 a.m. This changed to a first shift from 8:00 a.m. to 4:00 p.m., and a second from 4:00 p.m. until midnight. Individual workers’ working hours dropped to 7 from 9.5 per day on weekdays (Table S1). The workers had a 1-week shift rotating cycle; therefore, shift workers work on the day shift for 1 week and then switch to the night (or evening) shift for the next week. Although all the workers experienced decreased total working hours, only shift workers experienced the elimination of overnight work. Along with the introduction of a new shift system, a salary system was modified to prevent a drop in wages for workers.

### 2.2. Study Population

**Figure 1:**
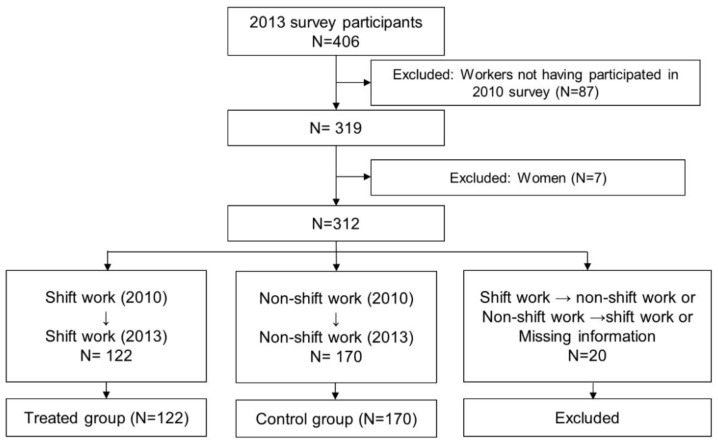
Flowchart of study subjects.

This study was based on data from the “risk assessment of musculoskeletal disorder”, which is a nationwide mandatory risk assessment for the prevention of work-related musculoskeletal disorders, investigating the risk factors and symptom complaints among workers [10]. Companies with risk factors for musculoskeletal disorders are required to perform this risk assessment every 3 years under the Occupational Safety and Health Act. The survey was performed in 2007, April 2010 (i.e., prior to the introduction of the new shift system in September 2010), and 2013. Out of 476 manual workers in Doowon, 406 workers participated in the survey in 2013. Individuals with no data from 2010 were excluded (*N* = 87), as well as female workers (*N* = 7). Shift workers in both 2010 and 2013 (*N* = 122) were considered as the treated group, while non-shift workers in both 2010 and 2013 (*N* = 170) were considered as the control group. The analytic sample selection process is depicted in Figure 1. All subjects gave their informed consent for inclusion before they participated in the study. The study was conducted in accordance with the Declaration of Helsinki.

### 2.3. Measures

Musculoskeletal pain (MSP) was assessed by a questionnaire administered to employees in groups of 50–60 during a prescheduled, 1-h session of “occupational safety and health education” guided by one of our researchers during workhours. The questions were derived from the National Institute for Occupational Safety and Health (NIOSH) symptom survey [16,17]. We modified the questionnaire slightly by combining the response options. The participants were asked: “Have you had any symptoms of pain, aches, stiffness, burning, numbness, or tingling in the following body parts over the past year?” Options for the specific body parts included neck, shoulder, arm/elbow, wrist/hand, back, and leg/foot. If they answered “yes”, then the frequency and duration of these symptoms were assessed. Response options for frequency of symptoms were as follows: daily, once a week, once a month, and every 2–3 months or more. Options for duration were as follows: less than 1 week, 1 week to 1 month, 1 month to 6 months, and 6 months or longer. If the participants answered that they had symptoms lasting for more than 1 week or occurring at least once a month within the past year, they were defined as having MSP.

Information on age, sex, education, shift work, weekly working hour over the past month, and salary in the last year were collected from the questionnaire. In 2010, risk factors for work-related musculoskeletal disorders were assessed by quick exposure check (QEC) [18]. This tool assesses physical workplace factors such as posture, movement, and handling weight and provides exposure level for 4 body regions by scoring system.

### 2.4. Statistical Analysis

We compared baseline demographic and occupational characteristics between the treated group (shift workers) and the control group (non-shift workers). Using generalized estimating equation (GEE) models, we estimated MSP rate changes in the post- to pre-intervention (introduction of a new shift system) between the treated and the control group. For difference-in-difference analysis, the GEE models included interaction terms between the time (pre vs. post) and the group (treated vs. control). Age and weekly working hours were included in the GEE model as covariates. The significance level for statistical analyses was *p* < 0.05 using a two-tailed test. SAS version 9.4 (SAS Institute, Cary, NC, USA) was used for statistical analysis.

### 2.5. Parallel Trends Assumption

The parallel trends assumption, which is critical for the internal validity of the DID framework, is that the treated group and control group display a parallel trend over time before the introduction of the intervention. To explore the validity of this assumption, we explored temporal changes in musculoskeletal outcomes prior to treatment from 2007 to 2010 (Figure S1). Note that the number of participants in 2007 was smaller than the samples underlying our main analysis because 66 individuals in the study sample did not participate in the 2007 survey. Graphical displays of the raw data presented similar trends in outcomes among the treated and control group over time, especially for shoulder and leg pain. We therefore conclude that we found no evidence for violation of the parallel trends assumption.

## 3. Results

The baseline characteristics of the study sample by treated and control groups are summarized in Table 1. The average age and working years were higher in the control group than in the treated group by around 3 years. Incomes were higher in the treated group, but education and weekly working hours were not significantly different between the two groups. Physical workplace factors assessed by QEC showed no significant differences between the two groups.

To display the change in MSP for workers, we have presented the percentages of individuals with MSP in Table 2. MSP prevalence for all sites combined was increased by 0.8% in the treated group and 11.8% in the control group, while pain in the upper extremities was reduced by 5.7% in the treated group but increased by 10.6% in the control group. After controlling for age and weekly working hours, the prevalence of MSP for shoulder (−10.3%), arm (−12.9%), all sites combined (−9.2%), and upper extremity combined (−14.8%) showed significant decreases from pre- to post-intervention among the treated group compared to the control group.

## 4. Discussion

The findings of this quasi-experimental study suggest a positive impact of decreased night work, as the prevalence of MSP in shift workers was decreased in comparison to non-shift workers after the implementation of a shift system change involving the elimination of overnight work. Considering that there were no differences in the baseline characteristics of shift workers and non-shift workers, including socioeconomic status or work characteristics such as physical workplace risk factors, as well as no evidence for violation of the parallel trends assumption, decreased MSP prevalence of shift workers was likely related to reduced night work.

This finding is in line with a number of previous studies. Some, but not all, cross-sectional studies have reported that shift work was associated with increased musculoskeletal diseases or symptoms [4,5,7,12,19,20]. A longitudinal study in a cohort of nurses found that the risk of lower back pain was 1.15 times higher in shift workers compared to day workers [13]. A prospective cohort study of Swedish twins reported an association between night work and disability pension due to musculoskeletal diagnoses (Hazard ratio [HR] 1.33 for 1–10 years of night work and HR 1.39 for over 10 years of night work) [6]. By contrast, several studies found no significant associations between night or shift work and musculoskeletal problems [21,22,23,24]. However, the majority of previous studies on night shift work and musculoskeletal problems were based on observation of a small number of subjects, and the measure of shift work and outcomes varied across studies.

Although the findings on the association between shift work and musculoskeletal disorders are not conclusive, the probable mechanism can be explained by lack or poor sleep due to shift work. Sleep deprivation increases catabolic hormones such as cortisol, which stimulate protein degradation; therefore, muscle recovery after exercise and injuries would potentially be compromised [25]. In addition, it is suggested that circadian rhythms may control the health and disease of the musculoskeletal system by influencing physical activity, feeding/fasting, body temperature, or hormonal/neuronal control [26]. In a number of observational studies, sleep was associated with musculoskeletal outcomes [8,9,27,28,29,30].

The psychosocial stress associated with night shift work may also play a role in the association between night work and MSP. Several studies have shown that psychosocial stress can be more significant for shift workers compared to day workers [31]. Occupational stress has been found to be associated with musculoskeletal outcomes in various settings [32,33].

In our data, the most significant changes after the new shift system between treated and control groups were found in the MSP prevalence of shoulder and arm. Work-relatedness of musculoskeletal disorders of upper extremities has been well established [34]. Considering that the majority of the Doowon plant workers were exposed to repetition, awkward posture, and mechanical force acting on the upper limbs (including shoulder and elbow) on the conveyor belt, it seems plausible that MSP of upper extremities would be most impacted by the intervention.

A strength of the study was that a follow-up period of 3 years was used to observe the difference before and after the change of the shift system. The time span within which an impact on MSP can occur is not known. However, most previous studies investigating the effect of ergonomic interventions for musculoskeletal disorders of the upper limb and neck among office workers involved much shorter follow-up periods of 12 months or less, and the results have been inconsistent [35]. There might be a possibility that the musculoskeletal effect of decreased night work had not yet occurred, or it had already faded away.

This study also has several limitations. First, the outcomes used in the study were self-reported pain, not clinically diagnosed musculoskeletal disorders; therefore, the results should be interpreted with caution, as there may be misclassification. However, self-reported MSP has been extensively used in previous epidemiologic studies of work-related musculoskeletal disorders and has been found to correlate well with physical findings from physical exams, ergonomic task characteristics, as well as compensation claims [36].

Second, unmeasured confounding could be an issue in our study. Although we observed the same individuals in each group over time, we may have missed time-varying factors influencing MSP, which differed between treated and control groups. For example, changes in leisure time physical activity (sports) or having a second job may have adversely impacted MSP.

Lastly, regarding the external validity of the current study, the participants of this study were full-time manual workers in the manufacturing industry. Mechanisms relating night shift work to MSP likely differ for service workers or office workers. In addition, the working hours or night work hours of study participants before the intervention were much longer compared to Western countries; hence, the results cannot be generalized to other countries, especially ones where working hours are not as long.

## 5. Conclusions

To our knowledge, this is the first study to evaluate a quasi-experimental intervention to decrease night work with observational data related to MSP. In the present study, shift workers showed better improvements in the musculoskeletal symptoms after the intervention of reducing working hours compared with non-shift workers. Our results suggest that decreasing night work can be considered as a form of organizational intervention to improve musculoskeletal symptoms for manual shift workers who are exposed to physical risk factors in the workplace.

## Figures and Tables

**Table 1:** Characters of study subjects.

	Treated (*N* = 122)		Control (*N* = 170)		*p*
	Mean ± SD	*N* (%)	Mean ± SD	*N* (%)	
Age	42.2 ± 4.5		45.2 ± 4.8		<0.0001
Education					0.50
<High school		0 (0.0)		4 (2.4)	
High school		98 (80.3)		125 (73.5)	
>High school		23 (18.9)		35 (20.6)	
Missing		1 (0.8)		6 (3.5)	
Working years	18.6 ± 4.2		21.2 ± 3.9		<0.0001
Income (KRW 10,000 per year)	5273 ± 803 (2010)		5092 ± 702 (2010)		0.07
	6246 ± 666 (2013)		6307 ± 694 (2013)		0.45
Weekly working hours *	52.6 ± 8.8 (2010)		51.6 ± 9.3 (2010)		0.39
	45.9 ± 3.4 (2013)		45.6 ± 3.0 (2013)		0.39
QEC—back	23.0 ± 8.4		25.1 ± 9.2		0.06
QEC—shoulder/arm	27.4 ± 9.1		29.2 ± 9.2		0.13
QEC—wrist/hand	23.4 ± 8.0		24.6 ± 8.0		0.23
QEC—neck	9.2 ± 4.2		9.6 ± 4.3		0.44

Age, education, and working years are based on 2010 results. * including overtime and weekend work. QEC; quick exposure check.

**Table 2:** Difference-in-difference analysis results for the prevalence of musculoskeletal symptoms.

	Pre	Post	Difference (Post–Pre)	DID (Unadjusted)	DID (Adjusted) ^a^
Body Site	%	%	%	% (95% CI)	% (95% CI)
Neck					
Treated	48.4	47.5	−0.8	5.1 (−6.0, 16.1)	5.4 (−6.3, 17.0)
Control	50.6	44.7	−5.9		
Shoulder					
Treated	54.9	50.8	−4.1	−11.8 ** (−23.3, −0.2)	−10.3 * (−22.2, 1.6)
Control	51.8	59.4	7.7		
Arm					
Treated	30.3	21.3	−9.0	−13.7 ** (−25.1, −2.3)	−12.9 ** (−24.7, −1.2)
Control	24.1	28.8	4.7		
Hand					
Treated	42.6	37.7	−4.9	−7.9 (−20.0, 4.3)	−5.9 (−18.2, 6.5)
Control	31.2	34.1	2.9		
Back					
Treated	48.4	53.3	4.9	4.3 (−8.2, 16.9)	6.4 (−6.7, 19.5)
Control	47.7	48.2	0.6		
Leg					
Treated	36.1	29.5	−6.6	−8.9 (−20.6, 2.8)	−8.9 (−21.1, 3.2)
Control	27.7	30.0	2.4		
All sites ^b^					
Treated	80.3	81.2	0.8	−11.0 ** (−20.1, −1.8)	−9.2 ** (−18.3, −0.2)
Control	71.8	83.5	11.8		
Upper extremities ^c^					
Treated	70.5	64.8	−5.7	−16.3 ** (−27.1, −5.5)	−14.8 ** (−25.9, −3.8)
Control	60.0	70.6	10.6		

* *p* < 0.1; ** *p* < 0.05. a: adjusted for age and weekly working hours. b: symptoms at neck, shoulder, arm, hand, back, or leg. c: symptoms at shoulder, arm, or hand.

## Supplementary Materials

The following are available online at https://www.mdpi.com/1660-4601/17/23/9092/s1. Table S1. Change of work schedule by treated and control groups. Figure S1: Prevalence of musculoskeletal pain in 2007 (*N* = 226), 2010 (*N* = 292), and 2013 (*N* = 292) among the treated and control groups.
