# Supplementary material for: Impact of Decreased Night Work on Workers’ Musculoskeletal Symptoms: A Quasi-Experimental Intervention Study
Source: Int J Environ Res Public Health. 2020 Dec 5;17(23):9092. doi: 10.3390/ijerph17239092 (PMC7730522; doi:10.3390/ijerph17239092)
Supplement: Supplementary file 1 [file ijerph-17-09092-s001.zip › Supplementary figure 1.pdf]

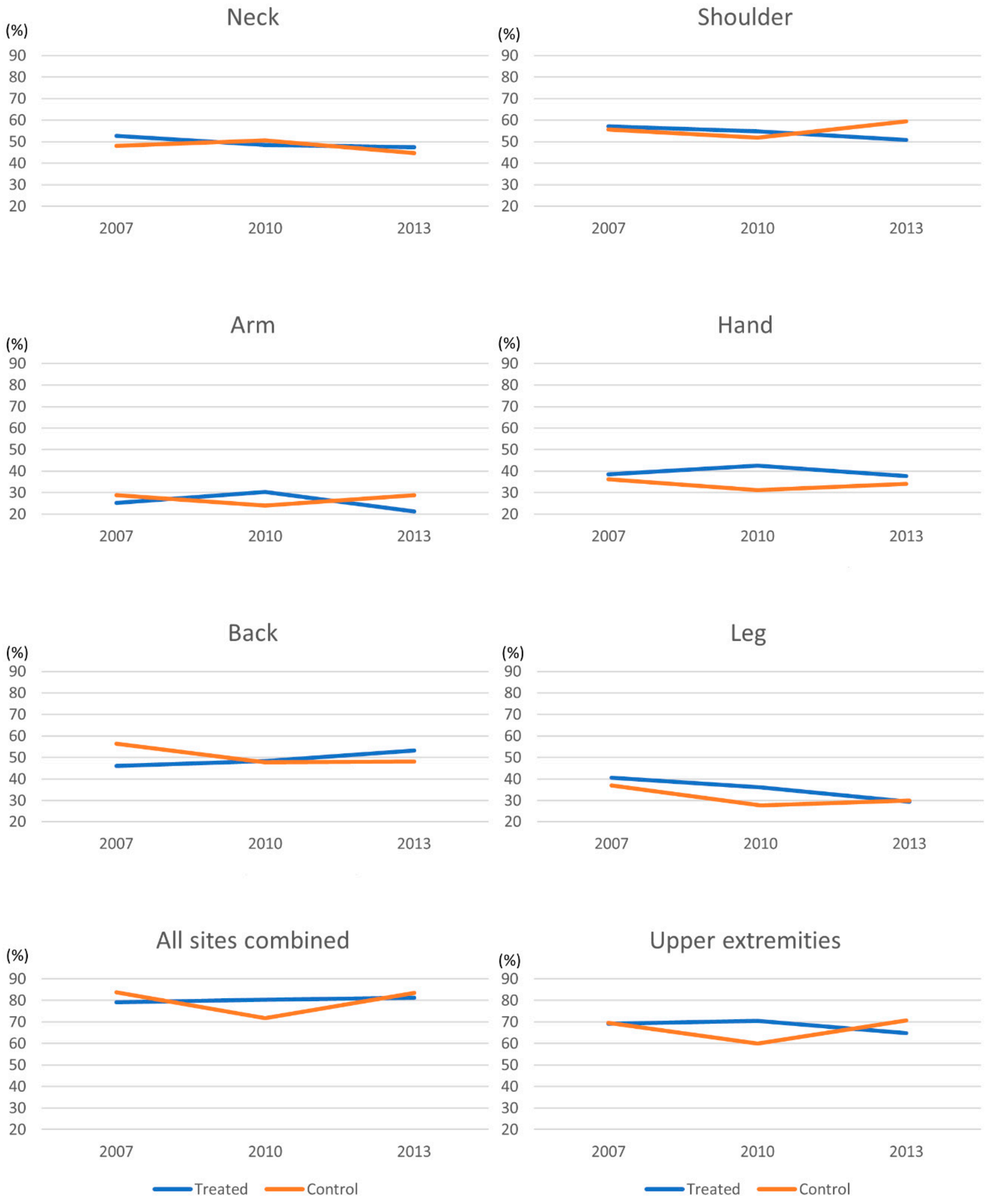

Supplementary figure S1 Prevalences of musculoskeletal pain in 2007 (N=226), 2010 and 2013 (N=292) among the treated and control group
