# Supplementary material for: Impact of Decreased Night Work on Workers’ Musculoskeletal Symptoms: A Quasi-Experimental Intervention Study
Source: Int J Environ Res Public Health. 2020 Dec 5;17(23):9092. doi: 10.3390/ijerph17239092 (PMC7730522; doi:10.3390/ijerph17239092)
Supplement: Supplementary file 1 [file ijerph-17-09092-s001.zip › Supplementary Table S1.pdf]

Supplementary Table S1. Change of work schedule by treated and control group.

	Before (2010)			After (2013)		
	Time	Hours	Activity	Time	Hours	Activity
Treated (shift workers)						
Day shift	08:30 - 10:15	1hr 45min	Work	08:00 - 10:00	2hr	Work
	10:15 - 10:30	15min	Break	10:00 - 10:10	10min	Break
	10:30 - 12:30	2hr	Work	10:10 - 12:00	1hr 50min	Work
	12:30 - 13:20	50min	Break	12:00 - 12:40	40min	Break
	13:20 - 15:15	1hr 55min	Work	12:40 - 14:30	1hr 50min	Work
	15:15 - 15:30	15min	Break	14:30 - 14:40	10min	Break
	15:30 - 17:20	1hr 50min	Work	14:40 - 16:00	1hr 20min	Work
	17:20 - 17:30	10min	Break			
	17:30 - 19:30	2hr	Work*			
	working hour	9hr 30min		working hour	7hr	
Night/Evening shift	19:30 - 21:45	2hr 15min	Work	16:00 - 18:00	2hr	Work
	21:45 - 22:00	15min	Break	18:00 - 18:40	40min	Break
	22:00 - 24:00	2hr	Work	18:40 - 20:30	1hr 50min	Work
	24:00 - 00:50	50min	Break	20:30 - 20:40	10min	Break
	00:50 - 02:45	1hr 55min	Work	20:40 - 22:30	1hr 50min	Work
	02:45 - 03:00	15min	Break	22:30 - 22:40	10min	Break
	03:00 - 04:20	1hr 20min	Work	22:40 - 24:00	1hr 20min	Work
	04:20 - 06:20	2hr	Work*			
	working hour	9hr 30min		working hour	7hr	
Control						
(non-shift workers)	08:30 - 10:15	1hr 45min	Work	08:00 - 10:00	2hr	Work
	10:15 - 10:30	15min	Break	10:00 - 10:10	10min	Break
	10:30 - 12:30	2hr	Work	10:10 - 12:00	1hr 50min	Work
	12:30 - 13:20	50min	Break	12:00 - 12:40	40min	Break
	13:20 - 15:15	1hr 55min	Work	12:40 - 14:30	1hr 50min	Work
	15:15 - 15:30	15min	Break	14:30 - 14:40	10min	Break
	15:30 - 17:20	1hr 50min	Work	14:40 - 16:00	1hr 20min	Work
	17:20 - 17:30	10min	Break			
	17:30 - 19:30	2hr	Work*			
	working hour	9hr 30min		working hour	7hr	

* mandatory overtime.
